# Characterization of Structural Connectivity of the Default Mode Network in Dogs using Diffusion Tensor Imaging

**DOI:** 10.1038/srep36851

**Published:** 2016-11-25

**Authors:** Jennifer L. Robinson, Madhura Baxi, Jeffrey S. Katz, Paul Waggoner, Ronald Beyers, Edward Morrison, Nouha Salibi, Thomas S. Denney, Vitaly Vodyanoy, Gopikrishna Deshpande

**Affiliations:** 1 AU MRI Research Center, Dept. of Electrical & Computer Engineering, Auburn University, Auburn, AL, USA; 2 Dept. of Psychology, Auburn University, Auburn, AL, USA; 3 Alabama Advanced Imaging Consortium, Auburn University and University of Alabama Birmingham, AL, USA; 4 Psychiatry Neuroimaging Laboratory, Department of Psychiatry, Brigham and Women’s Hospital, Harvard Medical School, Boston, MA, USA; 5 Canine Detection Research Institute, Auburn University, Auburn, AL, USA; 6 Dept. of Anatomy, Physiology & Pharmacology, Auburn University, Auburn, AL, USA; 7 MR R&D, Siemens Healthcare, Malvern, PA, USA

## Abstract

Diffusion tensor imaging (DTI) provides us an insight into the micro-architecture of white-matter tracts in the brain. This method has proved promising in understanding and investigating the neuronal tracts and structural connectivity between the brain regions in primates as well as rodents. The close evolutionary relationship between canines and humans may have spawned a unique bond in regard to social cognition rendering them useful as an animal model in translational research. In this study, we acquired diffusion data from anaesthetized dogs and created a DTI-based atlas for a canine model which could be used to investigate various white matter diseases. We illustrate the application of this atlas by calculating DTI tractography based structural connectivity between the anterior cingulate cortex (ACC) and posterior cingulate cortex (PCC) regions of the default mode network (DMN) in dogs. White matter connectivity was investigated to provide structural basis for the functional dissociation observed between the anterior and posterior parts of DMN. A comparison of the integrity of long range structural connections (such as in the DMN) between dogs and humans is likely to provide us with new perspectives on the neural basis of the evolution of cognitive functions.

Diffusion tensor imaging (DTI) is a magnetic resonance imaging technique that measures the anisotropic diffusion of water molecules in tissues providing useful structural information about white matter and the orientation of neural tracts. DTI first came into existence in the mid-1980s^1^,^2^,^3^ and has been rapidly developing ever since. This technique is noninvasive and is now widely used for studying structural connectivity in healthy populations as well as in psychiatric and neurological diseases traditionally characterized by white matter deficits. Researchers have been using DTI to study human and monkey brains and more recently, to study rodent brains such as mice^4^. However, there have been only a handful of studies applying this powerful method to canines, despite the increased use of these animals in research^5^,^6^.

Dogs are an appealing species to study for biological, behavioral, and evolutionary reasons which have led to the explosion of research focused on dogs (for reviews see^7^,^8^). One of the intriguing reasons for this development is that the close evolutionary history between dogs and humans may have spawned a unique bond in regard to social cognition^9^. In this context, canine social cognition involves studying dog-human interactions to understand how selection pressures via domestication may have (or have not) shaped canine social cognitive abilities (for reviews see Kaminski *et al*.^10^ and Miklosi^11^). These abilities can be tested/observed in a variety of tasks that measure canine performance in the presence of human cues. Such tasks include manipulation of human speech (emotional and semantic content), presence (absence or not), and communicative gestures (gazing, pointing, body posture) that are passively processed or convey solutions to problems (e.g., emotion contagion, emotion discrimination, guesser-knower paradigm, object choice task, object search, responding to commands, strange situation test, unsolvable task^12^,^13^,^14^,^15^,^16^). For example, in the object choice task, dogs need to attend to a person’s point and/or gaze to successfully locate a hidden object. Of note, not only do dogs display joint attention with humans and respond to human pointing and gesturing but they do so better than nonhuman primates making them an excellent animal model for human social cognition^9^,^17^,^18^,^19^. From an evolutionary perspective, the canine advantage is either due to the domestication of dogs (from their predecessors, wolves), their close contact with humans during their daily lives, or is a residual mechanism present before the lineages split 97 million years ago. Unraveling the different phylogenetic and ontogenetic possibilities and their interaction can be solved via triangulating findings comparing the behavioral, anatomical, and functional neural mechanisms within and across species to understand their evolutionary origin. Canine DTI represents an important technique to explore the neural basis of the evolution of cognitive function and its anatomical correspondences. Additionally, establishing a DTI-atlas in canines will serve as an important measure to allow researchers to ask questions concerning how fiber tracts may change with experience^20^,^21^.

Studying the canine model might thus give us a perspective into the evolution of cognitive functions. Additionally, dogs serve as a large animal model, critical for translation of quantitative MRI methods to clinical populations^22^, share a common environment with their owners until their old age, and receive a good level of healthcare^23^. Furthermore, rabies has a long history of association with dogs and is almost never known to infect or to be transmitted to humans by small rodents such as rats, chipmunks or rabbits^24^. For these reasons dogs are an interesting species worth studying as animal models in translational research to study aging and its effect on disease progression as well as to investigate human diseases that affect white matter integrity such as neurological demyelinating conditions (e.g., Multiple Sclerosis)^22^, GM1 gangliosidosis, a fatal neurodegenerative lysosomal storage disease^25^ and Rabies, a virtually incurable disease^26^. Thus, non-invasive imaging of neural white matter tracts in the dog brain by using DTI could prove to be very useful.

There have been a few DTI studies conducted on canine models including an *ex-vivo* study that was conducted on euthanized canine brains to study the white matter fibers by analyzing the fractional anisotropy (FA) and directional maps computed using the DTI images acquired within first 2 hours after death^5^ and an *in vivo* study investigating feasibility of diffusion tensor tractography in a canine model for studying the cerebral white matter in anesthetized dogs^6^. However, to our knowledge, ours is the first study to date, to have investigated *in vivo* diffusion images of dog brains to provide us with group-wise FA color maps. Therefore, in this study, we aimed to build a canine DTI-atlas, and demonstrate the utility of the atlas by testing a hypothesis derived from previous work in our lab^27^.

With regard to the second aim of our study based upon a resting state fMRI study recently conducted on dog brains in our lab^27^, we aspired to test the hypothesis that the functional connectivity dissociation between the anterior and posterior parts of the default mode network (DMN) in the canine model possesses a structural basis.

To accomplish the aims of our study, we first acquired diffusion-weighted images (DWI) in anesthetized dogs to develop a DTI-based atlas of the dog brain for use in future studies by giving an insight into the structural connectivity inside the dog brain. Second, we illustrate the utility of the dog DTI atlas to test a specific hypothesis arising from our previous resting state functional magnetic resonance imaging (fMRI) study^27^. Specifically, in our previous study we observed that the anterior and posterior parts of the DMN seem to be dissociated, unlike in monkey and human brains^28^,^29^. Therefore, we hypothesized that DTI tractography, specifically in the dorsal cingulum connecting the posterior cingulate cortex (PCC) and anterior cingulate cortex/medial pre-frontal cortex (ACC/MPFC), could provide a structural basis for this dissociation in the form of weaker structural connection between these regions in dogs as compared to humans.

The DMN is an interconnected network of brain regions that is active when an individual is at rest and becomes less active when performing attentionally demanding tasks^30^,^31^. The network preferentially activates when an individual is daydreaming, engaged in self-retrospection and self-introspection^32^. The core brain regions of DMN, consist of the medial prefrontal cortex (MPFC), posterior cingulate cortex (PCC), inferior parietal cortex (IPC), inferior temporal cortex (ITC) and (para) hippocampal formation. The main hubs of the DMN are within the MPFC and along the posterior midline including PCC^32^. In the DMN, PCC has been proposed to be of special interest as it is the only region that directly interacts with all other DMN nodes^33^. The anterior regions (ACC/MPFC) of the DMN along with the medial temporal lobe facilitates flexible use of episodic memories and associations to construct self-relevant mental simulations. These regions have has also been implicated in judgment of others^34^. The above mentioned two subsystems converge on important nodes of integration including PCC. PCC is commonly associated with episodic memory retrieval^35^,^36^. Therefore, DMN is believed to be responsible for consciousness and self-referential processing and has been linked to human cognition^32^,^37^,^38^. Studies have demonstrated the existence of the DMN in humans and monkeys^28^,^29^, however there are conflicting reports on whether they exist in rodents^39^,^40^,^41^. Our resting state fMRI study conducted on dog brains indicated that the anterior and posterior parts of the DMN are dissociated in dogs^27^. Previously conducted research has illustrated that the DMN exhibits the highest overlap in its structural and functional connectivity^42^. Therefore, we believed that there might be a significant possibility of the existence of a structural basis for the observed dissociation in the DMN in dogs. We thus decided to investigate it in the anticipation of attaining an insight into the evolution of cognitive functions.

## Methods

All methods and experiments were approved by the Auburn University Institutional Animal Care and Use Committee. We confirm that all methods were performed in accordance with the relevant guidelines and regulations. DWI data were acquired using a 3T MAGNETOM Verio (Siemens Healthcare, Erlangen, Germany) MRI scanner from 23 anaesthetized Labrador retriever dogs (age range 12–60 months) using a human knee coil (serving as a dog head coil) and an EPI (Echo Planar Imaging) based diffusion sequence with the following parameters: TR = 3.6 s, TE = 95 ms, flip angle = 90 °, 128 × 128 acquisition matrix, 30 diffusion directions, b = 0 and 1000, voxel size = 3 × 3 × 3 mm^3^. Dogs were sedated and lightly anesthetized with intramuscularly administered xylazine (2.2 mg/kg) and ketamine HCL (11 mg/kg), respectively. Details regarding the imaging procedure can be found in our previous publication^27^ describing resting state fMRI analysis on a subset of the dog sample employed here. Data were preprocessed using the standard FMRIB’s Diffusion Toolbox (FDT, which is part of the FMRIB software library (FSL)^43^,^44^,^45^. Data were first brain extracted, and then corrected for eddy current induced distortions.

### DTI-based Atlas

Following eddy current correction^46^, diffusion tensors were fit to the corrected data, creating tensor and fractional anisotropy (FA) maps for each dog^43^. Individual FA maps were then registered to a high-resolution canine *ex vivo* template created in a previous study^47^ using FMRIB’s linear registration tool (FLIRT)^48^,^49^,^50^. The resulting transformation matrix, computed while registering FA maps to the template, was applied to the tensor maps for each dog, thus obtaining the vector data of each subject in the same standard space using the “vecreg” command line tool in FSL as described in ref. 51. By averaging FA maps as well as vector data in the template space for all the dogs, an average FA map and average color-coded tensor map were computed to reduce the inter-subject variability in canine brain.

### Diffusion Tractography

Diffusion parameters were modeled using the BEDPOSTX tool in FSL for the eddy corrected data, and then probabilistic tractography was implemented as described previously^52^,^53^,^54^ using the PROBTRACKX tool in FSL to estimate the likely connections between two regions of interest (ROIs). The following parameters were used for BEDPOSTX: number of fibers per voxel = 2, burnin period = 1000, number of jumps = 1250, ‘sample every’ = 25, model with sticks, gradient nonlinearities considered and ARD weight = 1. For PROBTRACKX, we used the following parameters: number of samples = 5000, curvature threshold = 0.2, step length = 0.5, and maximum number of steps = 2000. The ROIs were defined as 4 mm radius spheres in the PCC and ACC/MPFC. The ROIs were positioned based on the co-ordinates of the peak z-values in PCC and ACC/MPFC obtained from the group independent component corresponding to the DMN which was derived from an ICA analysis of resting state fMRI data in the current sample. Probabilistic tracking was achieved by creating a probability density function at each voxel on the principal diffusion directions. Tractography analysis was performed at the whole brain level and not confined to individual hemispheres. Connectivity probabilities were estimated between the ROIs by repetitive sampling of the streamlines through the probability distribution function. The ROIs were defined in the template/atlas space and then registered to each subject’s native space using the inverse of the previously computed transformation matrix. In this way, fiber tracts were calculated in the subject space, and then transformed to the template/atlas space by applying the appropriate transformation computed earlier. The ACC-PCC tracts of each subject were then binarized and compiled to create a group level probability map of the tracts for dogs with a subject count threshold in which the value of each voxel represents the number of subjects that had a tract passing though that voxel. The group level probability map of the ACC-PCC tracts was binarized and overlaid on the group-wise mean FA map computed earlier. A group-wise mean FA value was extracted from the ACC-PCC tracts by averaging the FA values for the voxels constituting the ACC-PCC group-level connectivity map.

## Results

**Figure 1:**
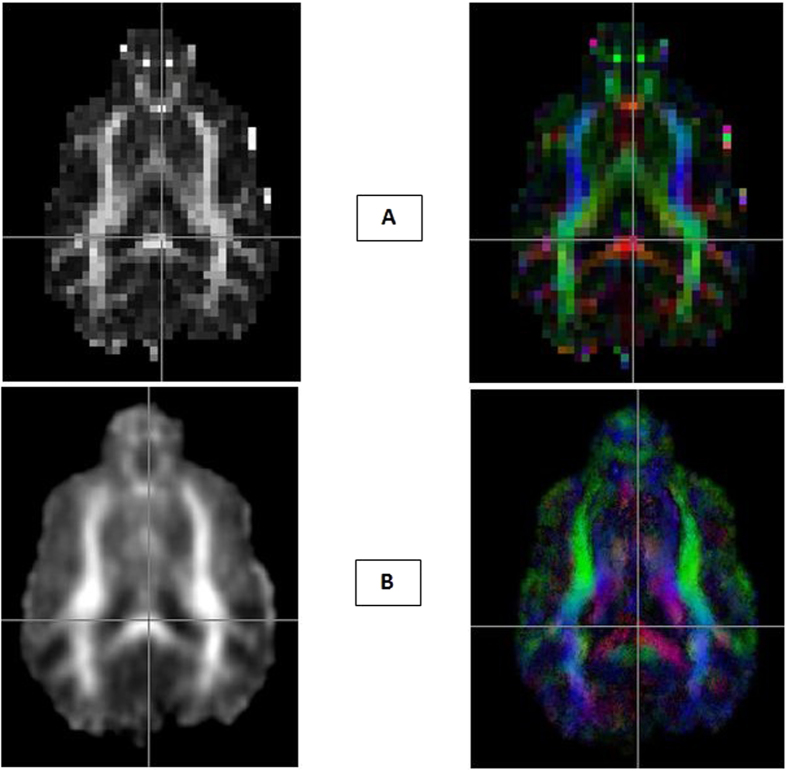
DTI Atlas. (**A**) FA map and Tensor based color map for one individual dog. (**B**) Average FA map and color map atlas using a group of 23 dogs. Color map represents left-right tracts in Red, anterior-posterior tracts in Green and superior-inferior tracts in Blue.

**Figure 2:**
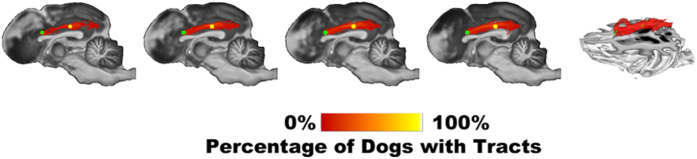
Group level probabilistic map of ACC-PCC tracts. The color scale indicates the percentage of dogs that had a tract in a given voxel. The green ROI corresponds to the ACC/MPFC while the yellow one shows the PCC.

Average FA and tensor-based maps were computed for individual dogs using the diffusion tensor computed from the acquired DWI data (Fig. 1A), and a group-wise average FA map with associated color map representing the first principal vector (i.e., the main diffusion direction) are shown in Fig. 1B. This provides a robust atlas void of disproportionate influence from any one subject. Fractional anisotropy values in the group FA map ranged from 0 to 1, with values closer to 1 in regions with higher anisotropy and more directional diffusion of water such as white matter, and closer to 0 in the areas of the brain with isotropic diffusion such as cerebrospinal fluid (CSF). Group mean FA was extracted from the ACC-PCC tract which was found to be 0.32 which is much lower compared to the mean FA previously calculated for humans (young adults)^55^, which was 0.57 (the caveats associated with this comparison are discussed in the next section). Additionally, the group level probability map of the tracts is shown in Fig. 2. The map shows the number of dogs having a tract passing through each voxel. Tracts connecting ACC to PCC were detected in only 9 out of 23 dogs. Even though, the tracts were detected in 9 of 23 dogs, the probability maps show voxel values less than 9 due to the inter-subject variability in the precise fiber pathways from one voxel to the next.

## Discussion

The average color map gives a qualitative characterization of the neural tracts in the white matter of a canine brain. The group-wise average FA map and tensor based map provides us with a DTI-based atlas for dogs by accounting for the inter-subject variability. This atlas can be utilized in future studies in both veterinary medicine and translational research. Readers can download the dog DTI atlas using the following link for such investigations: http://aucanlab.com/wp-content/uploads/2015/05/DogDTIAtlas.zip

As previously discussed, studying canines as an animal model in translational research could prove promising, keeping in mind that canines are phylogenetically distant from humans. Given their co-evolution with humans^1^,^9^,^17^ and capabilities to respond to human cues^17^,^18^,^19^,^56^, they are an interesting species worth studying to investigate how similar traits may have arisen via evolution. One brain network that has been implicated in cognition in general (as well as in social cognition)^57^,^58^ is the default mode network. The DMN is also known to underlie self-referential processing, consciousness, self-awareness and higher cognition in humans^32^,^37^,^38^.

A previous resting state fMRI study investigating the existence of the DMN in dogs, demonstrated disconnected ACC and PCC, the anterior and posterior regions of the DMN^27^. There is evidence that suggests that the DMN has a strong correlation between its structural and functional connectivity^42^. Therefore, in this study we tested the hypothesis that the functional finding about the anterior-posterior dissociation of the DMN in canines possesses a structural basis. The group mean FA for the MPFC/ACC-PCC tracts identified in the dog brain was notably low and also comparably lower than that observed in humans^55^. Additionally, the MPFC/ACC-PCC tract was found only in 9 out of 23 dogs indicating significant individual variability. As previously reported in a study conducted with humans^59^, tracts connecting MPFC/ACC to PCC were detected in 22 of 23 subjects. These observations lend support for our hypothesis that the weaker resting state functional connectivity found between the ACC and PCC in dogs compared to humans may have a structural basis.

Our results point toward certain hypotheses which can be tested in future studies. Given dogs perform better than nonhuman primates in several tests of social cognition, it raises questions about the centrality of the strong connection between the ACC and PCC found in humans and nonhuman primates for social cognition. In order to test related hypotheses, performance in behavioral tasks could be correlated with DTI-based metrics such as FA values. An additional advantage with DTI studies in animal research is that tract reconstruction can be compared with tracer studies to analyze corticocortical connectivity^60^. Such analyses could potentially give insight into individual canine performance as well.

While the current findings lend some support to convergent evolution (as opposed to homology due to the weak ACC and PCC connection in canines), it may well be that dogs are well equipped to learn the appropriate cues ontologically to perform well in social cognition tasks. Likely, it is an interaction between phylogenetic and ontogenetic factors that underlie the social cognition traits in dogs. The DTI-atlas can serve to allow researchers to ask questions concerning how fiber tracts may change with experience^20^,^21^. If such canine brain plasticity occurs, it could reveal an important piece of the puzzle as to how dogs become expert detector dogs, therapy dogs, and succeed in social cognition and other cognitive tasks. These aspects are worthy of future investigations.

### Limitations

While creating a DTI atlas for the dog brain is a clear contribution of the paper, direct comparison of FA values between dogs and humans for testing our specific structural hypothesis regarding the DMN involves certain caveats: (i) the size of the brain in dogs is smaller than in humans, but the spatial resolution of diffusion data acquired was not proportionally higher in dogs, (ii) the exact placement of seed ROIs in humans is likely to be more accurate than in dogs given that labeled atlases are available in humans, and (iii) the anatomy of dog and human brains, especially the frontal cortex, may not be exactly comparable. Further, the atlas itself is limited to canines of a specific breed (Labradors in this case) and age range. Larger studies in the future employing a larger sample of canines drawn from various breeds and age ranges are required to develop a more general canine DTI atlas. The current investigation is also constrained by the general limitations of diffusion tensor imaging, including spatial resolution, which lends itself to multiple fiber orientations being present in a single voxel (and thus, their contributions to values such as anisotropy may be averaged). Given that high diffusion anisotropy values depend on microscopic sources of anisotropy and macroscopic homogeneity within a voxel, some regions may be misrepresented as having no fibers (FA < 0.2), when really they just have multiple orientations. Relatedly, there is a vast data reduction that occurs in the analysis as algorithms typically assign 1 or 2 directions within a given voxel. Finally, DTI is sensitive to the motion of water which is relatively minor (~5–10 um), lending itself vulnerable to physiological artifacts that could interfere with DTI. Notwithstanding these limitations, our preliminary results regarding the anterior-posterior structural (as well as functional) dissociation in the canine DMN is worth exploration in the future for understanding the evolutionary significance of the DMN as well as the evolutionary bond between dogs and humans.

## Additional Information

**How to cite this article:** Robinson, J. L. *et al*. Characterization of Structural Connectivity of the Default Mode Network in Dogs using Diffusion Tensor Imaging. *Sci. Rep.* **6**, 36851; doi: 10.1038/srep36851 (2016).

**Publisher’s note:** Springer Nature remains neutral with regard to jurisdictional claims in published maps and institutional affiliations.
